# Unlocking the potential of iPSC-derived immune cells: engineering iNK and iT cells for cutting-edge immunotherapy

**DOI:** 10.3389/fimmu.2024.1457629

**Published:** 2024-08-30

**Authors:** Minggang Fang, Alexander Allen, Chong Luo, Jonathan D. Finn

**Affiliations:** Cell Therapy, Tome Biosciences, Watertown, MA, United States

**Keywords:** iPSC, iNK, iT, next generation immunotherapy, engineering strategies, differentiation

## Abstract

Induced pluripotent stem cells (iPSCs) have emerged as a revolutionary tool in cell therapies due to their ability to differentiate into various cell types, unlimited supply, and potential as off-the-shelf cell products. New advances in iPSC-derived immune cells have generated potent iNK and iT cells which showed robust killing of cancer cells in animal models and clinical trials. With the advent of advanced genome editing technologies that enable the development of highly engineered cells, here we outline 12 strategies to engineer iPSCs to overcome limitations and challenges of current cell-based immunotherapies, including safety switches, stealth edits, avoiding graft-versus-host disease (GvHD), targeting, reduced lymphodepletion, efficient differentiation, increased *in vivo* persistence, stemness, metabolic fitness, homing/trafficking, and overcoming suppressive tumor microenvironment and stromal cell barrier. With the development of advanced genome editing techniques, it is now possible to insert large DNA sequences into precise genomic locations without the need for DNA double strand breaks, enabling the potential for multiplexed knock out and insertion. These technological breakthroughs have made it possible to engineer complex cell therapy products at unprecedented speed and efficiency. The combination of iPSC derived iNK, iT and advanced gene editing techniques provides new opportunities and could lead to a new era for next generation of cell immunotherapies.

## 1 Introduction to immunotherapy

In the past decade, the field of cancer therapy has been significantly transformed by the advent of CAR-T cell immunotherapy, which has emerged as a pivotal treatment modality alongside traditional methods such as surgery, radiation, and chemotherapy. This innovative approach of engineering T cells with chimeric antigen receptors (CARs) to redirect T cells to recognize antigens such as CD19 or BCMA have shown remarkable efficacy against certain types of leukemia, lymphoma, and multiple myeloma (1). Since 2017, six CAR-T therapies have been approved by the U.S. Food and Drug Administration (FDA), with many more products at various stages of clinical development (2). CAR-T cells are also being explored for a multitude of nononcologic indications, including transplant rejection, infection, autoimmunity, cardiovascular disease, fibrosis, and senescence (3–6). However, the limitations of autologous FDA approved CAR-T therapies, such as cost, donor variability and time required for manufacturing, are widely recognized barriers to the wide adoption of CAR-T therapy (7–9). iPSCs have the potential to serve as a lower-cost source of high-quality engineered, off-the-shelf therapy, with scalable manufacturing and consistent product quality. To realize this vision and enable large-scale manufacturing, researchers have worked to develop and improve iPSC-to-T cell differentiation protocols.

Natural killer (NK) cells are another type of immune cell that can kill target cells via cytotoxic mechanisms. CAR-NK cells may have some notable benefits over CAR-T cells, such as: (1) increased safety (no neurotoxicity or cytokine release syndrome, and avoidance of GvHD), (2) several ways to trigger cytotoxic action, and (3) great likelihood of “off-the-shelf” production (7, 10). CAR-NK cells could be engineered to target a broad range of antigens, with the potential to deliver potent responses for cancer and autoimmune conditions (11, 12). The development of CAR-NK cells represents an exciting frontier in immunotherapy, with the potential to overcome the limitations of current CAR-T cell therapies and provide patients with more accessible and effective treatment options (7, 13).

Other immune cell types being studied for their potential in immune cell therapy are phagocytic cells, such as macrophages and dendritic cells. They patrol the body and assist in cleaning up infection and activating other immune cells. For solid tumors, macrophages can efficiently infiltrate into tumors and are abundantly present in tumor microenvironment (TME). As major immune regulators, CAR macrophage can turn cold TME (absence of T cells and pro-inflammatory cytokines) to hot (presence of T cells and pro-inflammatory cytokines) and attract and activate adaptive immune cells, in addition to directly killing tumor cells (14). As a result, there is a great interest in creating CAR macrophages for cancer immunotherapy in order to get around some of the problems that CAR T/NK treatment has, particularly with solid tumors (7, 14). Dendritic cells are a specialized type of phagocytic cell presenting antigen to bridges innate and adaptive immunity. Dendritic cells are crucial in the induction of immune responses to pathogens and tumors as well as for the maintenance of self-tolerance. Understanding the strengths and limitations of different cell types is highly needed to engineer cell therapies. The traits of CAR T, NK and macrophage are summarized in **Table 1**. Given that the current focus of cell-based immunotherapy are T cells and NK cells, we have focused the rest of this review on those two cell types.

**Table 1:** Characterization and comparison of current CAR T, NK and macrophage immunotherapies.

	CAR T	CAR NK	CAR Macrophage
Cell source	auto, allo, iPSC	allo, auto, iPSC	auto, iPSC
Off-the-shelf	no	yes	yes
Toxicities	CRS, ICANS common	less common	no clinical data
Cytotoxicity mechanisms	CAR-dependent	CAR-dependent and-independent	multiple, immunostimulatory TME
Infiltration into tumors	poor	poor	abundant
Clinical experience	Proven efficacy, 6 FDA approved	Limited but promising	very limited clinical experience

CAR, chimeric antigen receptor; auto, autologous; allo, allogenic; CRS, cytokine release syndrome; ICANS, Immune effector cell-associated neurotoxicity syndrome.

## 2 Generation and engineering of iPSC-derived iNK and iT cells

### 2.1 Overview of the generation process of iPSCs differentiation into iNK and iT cells

iPSCs are a type of stem cell derived from somatic cells, usually skin fibroblast or white blood cells by expression of Yamanaka factors (15, 16). Due to their unique features: unlimited expansion, the ability to differentiate into different cell types, and ease of editing, iPSCs have been developed as a new method to generate transplantable immune cells.

**Figure 1:**
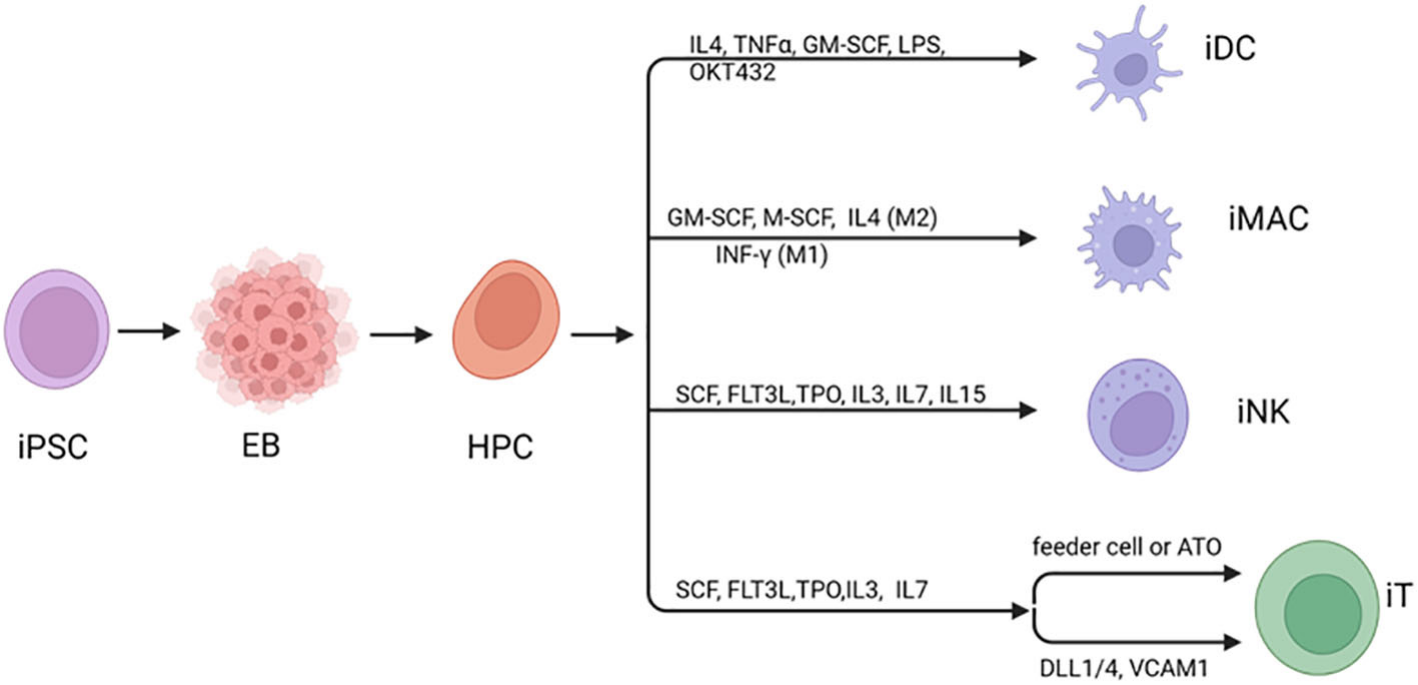
Differentiation of iPSC to immune cells. Human iPSC can be induced to differentiate to hematopoietic cells usually through generation of mesoderm in embryoid bodies (EBs). During differentiation to mature blood cells, hematopoietic progenitor cells (HPCs) are generated, leading to a diverse group of cells that can differentiate into various specialized cell types. Under specific cytokines and stimulating factors, HPC can be directed various immune cell types. Created with BioRender.com.

The differentiation of iPSCs to immune cells is a multiple step process. First, iPSCs undergo mesoderm induction in embryonic bodies (EB) upon the loss of pluripotency-related gene expression as well as increased expression of mesodermal genes to form multipotent progenitor cells. Second, hematopoietic progenitor cells (HPC) are induced and produce a population of cells capable of committing to various cell lineages (**Figure 1**). HPC are guided toward common lymphoid progenitors (CLP) cells, which subsequently develop into iNK or iT cells with a particular set of factors and extracellular matrix supports. As the focus cell type of this review, the procedures of iNK, iT generation are discussed in detail in following sections. In order to differentiate into iPSC-derived macrophages (iMACs), floating EBs are reseeded for attachment to culture vessels. This creates myeloid factories that give rise to progenitor macrophages, which then mature into M1 or M2 macrophages after incubation with specific cytokines (17, 18). For iPSC-derived dendritic cells (iDC), HPC are directed to CD11c+ DCs through exposure to a cocktail of growth factors (19, 20). These cell types are at different stages of research in preclinical and clinical settings and iNK cells are the most advanced and have demonstrated clinical efficacy in hematological malignancies.

### 2.2 Methods of iNK differentiation from iPSC

The differentiation of iNK cells from iPSCs is a complex but fascinating process which has evolved in the past 20 years. As was recently and excellently reviewed (21–23), a number of research groups have developed methods for producing NK cells from iPSC and embryonic stem cells (ESCs), including stromal cells and stroma-free protocols, or 2D and 3D differentiations.

In initial 2D studies from the Kaufman group, human ESCs were co-cultured with murine bone marrow (BM) stomal cells in cytokine-enriched media with BMP4, FGF2, VEGF, and SCF (24, 25). During the first step in differentiating iPSCs into iNK cells, hematopoietic lineage is induced to form hemogenic endothelium (HE). From the HE, CD34+ CD45+ hematopoietic progenitors are generated, then sorted and co-cultured with a second stromal feeder cells in medium containing interleukin IL-3 (during the first week), IL-7, IL-15, SCF, IL-2, and Flt3L (24, 26). Although Notch signaling is required for the stimulation of T cell development, it also plays a role in the effective development of NK cells in humans (27–29).

This 2D protocol was then modified to 3D, the “spin EB (embryoid bodies)” method. This method capitalizes on the fact that when iPSCs are cultured in suspension without feeder layers (stroma and serum free), they spontaneously form aggregates known as EB, before differentiation toward the NK-cell lineage (30–32). The “spin EB” protocol produces hematopoietic organoids that contain HPC, as well as endothelial and mesenchymal cells. These HPC then differentiate into NK cells under defined conditions (31, 32). This approach enhances the reproducibility and uniformity of the resulting iNK cells. IL-3 and IL-7 are commonly used to promote CLP which are a precursor to NK cells. From CLPs NK cell precursors begin to form in response to IL-15, which is required for proper differentiation and maintenance of NK cells. The emerging iNK cells are expanded and matured in culture. During this phase, they acquire the necessary receptors and functional capabilities that characterize mature NK cells, such as the ability to recognize and kill tumor cells. Feeder cells, such as K562 or EBV-LCL cell which are susceptible targets for NK cells, have been engineered with membrane bound IL-21 and 4-1BBL to improve expansion and cytotoxicity (33–36). Similar to PB-NK and UCB-NK cells, hESC/iPSC-derived NK cells express both activating and inhibitory receptors, including TNF-a, CD16a, NKG2D, TRAIL, NCR receptors (NKp44, NKp46), IFN-g, and NKG2D. The interplay of different cytokine combinations on NK cell differentiation and maturation are still being explored and warrant further research.

The differentiation of iNK cells from iPSCs represents a significant achievement in the field of regenerative medicine and immunotherapy. By leveraging the plasticity of iPSCs and the power of modern cell culture techniques, scientists are able to produce iNK cells that could potentially be used to treat a variety of cancers and autoimmune conditions. While still in its infancy, iPSC derived iNK cells from multiple groups are already showing promise in the clinic, where they have demonstrated deep depletion of target B cells in oncology, and currently being evaluated in autoimmune diseases (12, 37).

### 2.3 Methods of iT differentiation from iPSC

The stem cells used to make iPSC-derived T cells can come from different types of somatic cells. The iPSC derived T cells express random TCR due to rearrangement after differentiation from non-T cells that contain germline TCR genes. Cell therapy can be accomplished by introducing the exogenous TCR into iPSCs. Transgenic TCRs generate the CD3 signal during T cell differentiation, which inhibits the rearrangement of endogenous TCRs, allowing T cells derived from iPSCs to target specific antigens (38). Studies to engineer exogenous TCRs to generate potent T cells from iPSCs have produced exciting results (38, 39). Reprogramming of iPSCs can also be derived from peripheral blood T cells. In this case, the rearranged TCR gene is retained in iT cells. Antigen-specific T cell clones can be reprogrammed to produce antigen-specific T cells, or T-iPSCs can be engineered with a CAR to improve tumor specificity for use in cell therapy. The only iT cell candidate in clinical trials is FT819, derived from T-iPSC cells, where TRAC is knocked out and a CD19 CAR is inserted into iPSC before differentiation into iT cells (40). The results of phase 1 clinical trial are promising: no dose related toxicity, no ICANS or GvHD, no grade 3 CRS, and evidence of anti-tumor activity, with 3/15 patients demonstrating complete response (41).

iT cell differentiation from iPSCs has been historically challenging. The most challenging part of iPSC derived T cells is how to generate potent iT cells from iPSC in serum and stromal cell free conditions. For the natural T cell development process *in vivo*, CD34+CD43- hemogenic epithelial (HE) cells at AGM (Aorta-ganod-mesonephros) undergoes endothelial-to-hematopoietic transition (EHT), in which the HE is rounded up and releases the floating cells with HPC markers CD34 (42, 43). In a process dependent on Notch signaling, HPC differentiate into CD5/CD7 double positive T cell progenitor cells. Under reduced Notch signaling and increased TCR signaling, CD4/CD8 double positive cells are produced and mature to single positive T cells (44–46). This understanding of endogenous T cell differentiation has informed multiple efforts (reviewed below) to recreate the T cell differentiation *in vitro*.

The Zuniga-Pflucker group at Toronto University has done seminal work to recreate T cell development *in vitro*, by expressing Notch ligand DL1, DL4 in thymic stromal cells (47). Subsequent feeder-based systems, such as the artificial thymic organoid (ATO) platform, have been developed to better replicate the three-dimensional structure of the thymus (39, 48). The latest breakthrough is chemically defined differentiation with DL4 and VCAM1 in feeder cell free condition (44, 45, 49, 50). The stromal cell-free, DL4 microbead-based approach that supports efficient *in vitro* development of human progenitor T cells from pluripotent stem cells (PSCs), provides a simple, robust and potentially scalable platform to both study human T cell development and facilitate the development of engineered T cell therapies from renewable sources (50).

All the pioneering groups started the same iT differentiation process from iPSCs with EB formation and isolated CD34 HE cells from EB. The major difference was the different coating matrix. Kaneko group seed HPCs on to plates coated with recombinant DL4 and retronectin, a fragment of the fibronectin protein that can also bind to integrin as VCAM1. This process yielded CD8 single positive (CD8SP) cells that expressed the original TCR, but they failed to generate iT cells from non-T iPSC lines where the TCR locus is unrearranged (49). This limitation indicates that this protocol fails to capture some key aspects of T cell development. A major milestone was from the Zandstra group, which showed synergistic effect of VCAM1 and DL4 to enhance Notch signaling and progenitor T cell differentiation (51). This group demonstrated that primary human HPC from CB could be directed to become T cell progenitors on DL4, VCAM1 coated plates and mature T cells *in vivo* with T cell progenitor transplantation. VCAM1 is a stromal matrix protein and a ligand for integrin which is highly expressed in HPC cells. Closely following Kaneko’s work, the Zandstra and Daley group finally differentiate iPSCs to mature T cells in a feeder free system in 2022. They isolated CD34 positive HE cells from EB, seeded them on DL4+VCAM1 coated plates, grew them with SCF, TPO, IL-3, IL-7 and TNFa, for another 14 days, resulting in CD5CD7 double positive cells. The addition of VCAM1 significantly increased the percentage of CD5CD7 double positive progenitor cells, and TCRαβ, γδT cells were generated with diverse TCR repertoire from TCR locus sequencing (44, 45).

### 2.4 Limitations of iPSC derived therapeutics

While iPSCs hold great promise for regenerative medicine, there are a few potential limitations that need to be addressed. First, iPSCs can be prone to genetic instability, including point mutations, copy number variations, and chromosomal rearrangements that can result from donor somatic cells, during reprogramming, or during extended cell culture (52, 53). Residual undifferentiated iPSCs have the potential to form teratomas or other types of tumors if transplanted into patients (54). During the iPSC creation and differentiation process, routine genetic screening and monitoring are carried out utilizing high-throughput sequencing and other cutting-edge genomic technologies to identify and remove cells with genetic abnormalities. Advanced differentiation techniques can be developed to ensure that all iPSCs fully differentiate into the intended cell types, and the final differentiated cell products can be purified with selection in order to remove residual iPSCs. Furthermore, as discussed in section 3.1, suicide genes/safety switches can be inserted into iPSCs and activated to eradicate any undifferentiated iPSCs that may still exist, further improving the safety profile of iPSC derived cells.

## 3 iPSC engineering for next generation iNK, iT cells

**Figure 2:**
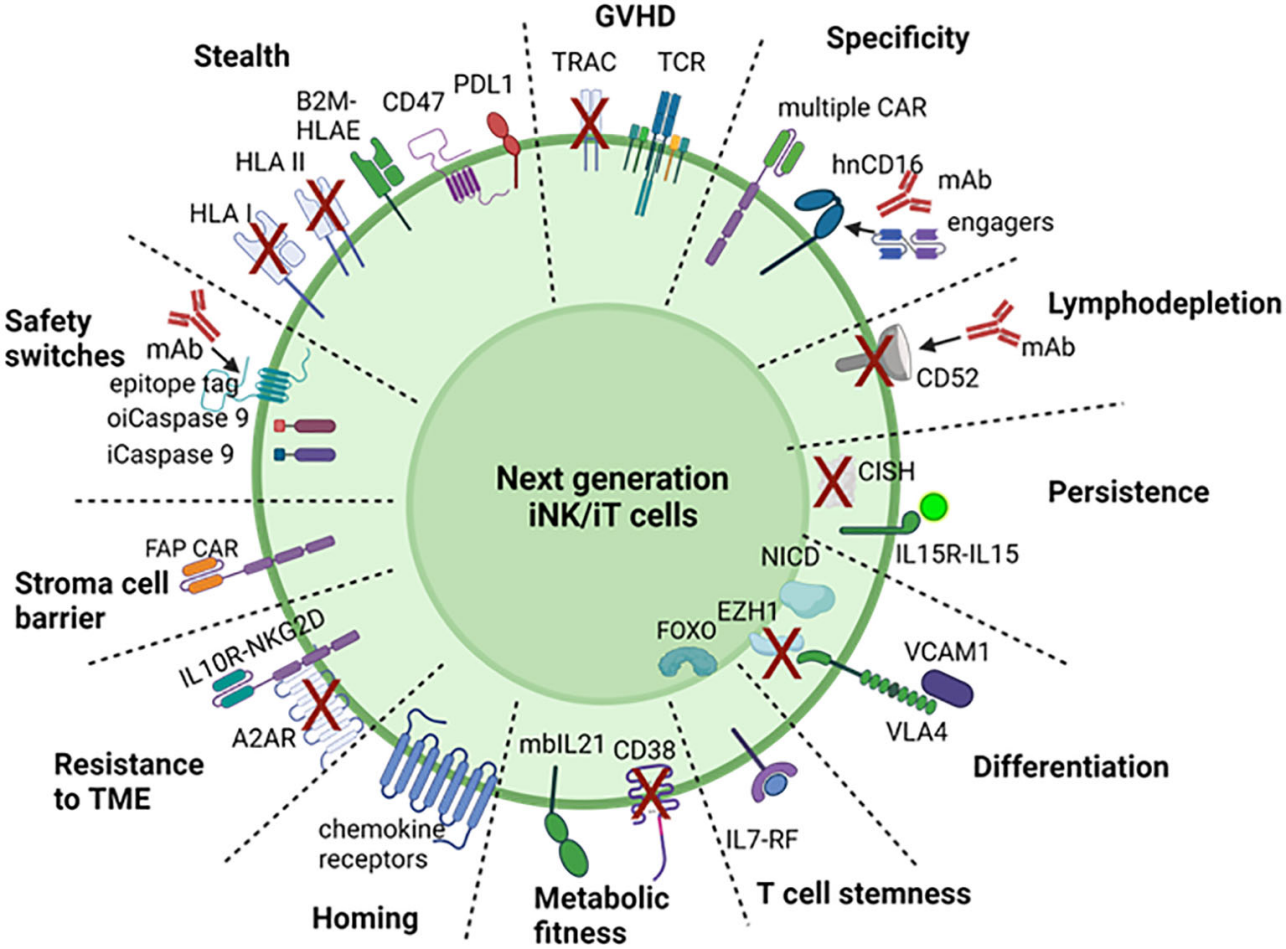
Engineering strategies for next generation iNK, iT cell. To overcome the challenges and limitation of CAR T or NK cell for immunotherapy, 12 strategies are proposed to engineer iPSC for with safety switches, stealth edits, specific targeting, avoiding GvHD, lymphodepletion, *in vivo* persistence, efficient differentiation, T cell stemness, metabolic fitness, homing/trafficking, overcoming suppressive tumor microenvironment and stromal cell barrier. OiCaspase 9, orthogonal inducible caspase 9; iCaspase 9, inducible caspase 9; GvHD, graft versus host disease; KO, knockout; KI, knockin; hnCD16, high affinity non-cleavable CD16; mIL-15RF, membrane bound IL15-receptor fusion; NICD, notch intracellular domain; Flt3L, FMS‐like tyrosine kinase 3 ligand; IL-7RF, IL-7 receptor fusion; ICI, immune checkpoint inhibitor; DNTGFbR, dominant negative TGFβ receptor; IL10DR, IL-10 decoy receptor; FAP, fibroblast activated protein. Created with BioRender.com.

FDA approved T cell therapies have had a remarkable impact on patient care for a subset of hematological malignancies. This foundation has motivated the development of off-the-shelf engineered T and NK cell therapies for a broad range of indications. Achieving this vision will require cost-effective manufacturing of precision cell products capable of addressing multiple process and clinical-design challenges. In addition, expanding the breadth of indications possible with cell therapy and ensuring highly effective and safe therapies will require sophisticated cell engineering. For next generation iT, iNK cell therapies, the strategies outlined below could overcome current challenges and limitations of iPSC derived therapies and lead to better success of immune cell therapies, summarized in **Figure 2** and **Table 2**.

**Table 2:** Summary of edits for engineering iNK, iT cells.

Strategies	Edits	Examples
Safety switches	OiCaspase 9, iCaspase 9, epitope-Ab targeting	iCaspase 9 (55, 56)
Stealth edits	B2M, CIITA DKO +B2M-HLAE, CD47 KI	B2M KO (68–73)
GvHD	TRAC KO or antigen specific TCR KI	TRAC KO (98–100)
Specificity	Multiple CAR, hnCD16 KI	CAR (101–103)
Lymphodepletion	CD52 KO for CD52 Ab conditioning	Not available
In vivo persistence	mIL-15RF, CISH KO	IL-15 KI (121) CISH KO (127)
Differentiation/expansion	NICD, EZH1 KO, Flt3L, BMP4	EZH1 KO (45)
T cell stemness	IL-7RF KI, FOXO KI	FOXO KI (114)
Metabolic fitness	CD38 KO, mbIL-21	CD38 KO (23, 130)
Homing/infiltration	Chemokine receptors KI	CXCR4 KI (147)
Suppressive TME	ICI PD1, CTLA4, LAG3 decoy receptor, DNTGFbR, IL10DR KI	DNTGFbR (154–156)
Stromal cell barrier	FAP CAR	FAP CAR (169)

OiCaspase 9, orthogonal inducible caspase 9; iCaspase 9, inducible caspase 9; GvHD, graft versus host disease; KO, knockout; KI, knockin; hnCD16, high affinity non-cleavable CD16; mIL-15RF, membrane bound IL-15-IL-15 receptor fusion; NICD, notch intracellular domain; Flt3L, FMS‐like tyrosine kinase 3 ligand; IL-7RF, IL-7-IL-7 receptor fusion; ICI, immune checkpoint inhibitor; DNTGFbR, dominant negative TGFβ receptor; IL10DR, IL-10 decoy receptor; FAP, fibroblast activated protein.

### 3.1 Safety switches

Safety switches provide a mechanism to eliminate the infused cells in case of adverse events, thus increasing the safety of these therapies. There are several types of safety switches used in cell therapy as summarized in **Table 3**.

**Table 3:** Summary of different safety switch types.

Type	Characterization	Advantage	Disadvantage	References
HSV-TK	Express HSV-TK kinase and in combination with antiviral drug ganciclovir	Ganciclovir clinical available; confirmed safety	Only work in dividing cells;immunogenicity; Not suitable to CMV patients	(63, 66)
Antibody-based targeting	Express an epitope at the engrafted cell surface in combination with antibody targeting	Clinical available antibodies	Slow and incomplete ablation with low epitope expression	(58–61)
iCaspase 9	Inducible caspase suicide gene combination with bio-inert small molecule	Rapid; Human protein no immunogenicity	Potential iCasp9 resistance	(55–57)

HSV-TK, herpes simplex virus thymidine kinase; iCaspase 9, inducible caspase 9.

Inducible apoptosis: This method involves a safety switch based on the fusion of human caspase 9 to a modified FK-binding protein, which can be activated by a synthetic dimerizing drug (55, 56). When the drug is administered, it induces apoptosis in the cells expressing this construct, effectively eliminating them. This approach has been clinically validated, where the Inducible Caspase 9 (iCaspase-9) safety switch allowed for the rapid elimination of more than 90% of the modified T cells within 30 minutes after administration of a dimerizing drug, effectively ending GvHD without recurrence (57). The iCaspase-9 system is advantageous because it’s derived from human proteins, reducing the potential for immunogenicity. It also doesn’t rely on DNA synthesis for its activity, meaning it can control both dividing and non-dividing cells. This makes it a valuable tool for improving the safety of various cell therapies.

Antibody-mediated cytotoxicity: In order to destroy the therapeutic cells, monoclonal antibodies are used to target particular antigens on their surface. Unlike small-molecule targeting, T cell expression of cell surface markers allows for targeted elimination through the administration of target-specific antibodies, which also allows for monitoring by flow cytometry. This strategy includes the use of truncated human epidermal growth factor receptors (58, 59), RQR8 (60), CD20 (61), and permits the clearance of specific cell populations (expressing the specific receptors) for elimination by administration of FDA-approved antibodies like cetuximab or rituximab. In preclinical models this approach has the potential drawback of slow and insufficient cell ablation that have poor receptor expression (62).

HSV-TK suicide gene: HSV-TK (herpes simplex virus thymidine kinase) based suicide switch is a safety feature that has been previously used in cell therapy for control of GvHD in patients (63), particularly in the context of adoptive cell transfer like T-cell. HSV-TK is a cell cycle-dependent suicide gene, that catalyzes the generation of triphosphate ganciclovir (GCV), which is toxic to proliferating cells by inhibiting DNA chain elongation (64–66). In addition to cell-cycle dependence, another limitation of HSV-TK is the immunogenicity of the viral TK protein. Various clinical studies with HSV-TK transduced donor lymphocyte have been conducted, confirming its safety and efficacy (63, 66).

These safety switches are designed to manage potential side effects associated with advanced cellular therapies and expand their clinical applications by providing a controlled way to eliminate therapeutic cells if necessary.

### 3.2 Stealth edits

Because of its potential cost-effectiveness, scalability, and on-demand availability, allogeneic cellular immunotherapies hold considerable promise for the treatment of cancer and autoimmunity. A significant barrier to achieving therapeutic responses similar to those seen with existing autologous CAR-T cell treatments is immunological rejection of adoptively transferred allogeneic T and NK cells. For immunotherapy, four different kinds of genetic modifications for immune evasion have been investigated (**Table 4**). Human Leukocyte Antigens (HLA) genes play a crucial role in graft rejection. Differences between the recipient’s and graft’s HLA class I and II genes can lead to activation of host CD8+ and CD4+ T cells, respectively, resulting in direct killing of graft cells. Researchers are investigating ways to modify HLA expression on T or NK cells to be immunologically silent and evade the host versus graft (HvG) response, sometimes termed “stealth”.

**Table 4:** Features of stealth editing strategies in cell therapy.

Type	Characterization	Advantage	Disadvantage	References
HLA depletionand HLA-E/G-B2M KI	Direct remove the major HLA mismatch	Reduced CD8, CD4 T and NK cell recognition	None reported	(68–73)
Immune checkpoint inhibitor	Overexpress ICI at cell surface	Simple and easy engineering	None reported	(81, 88, 89)
ADR	CAR targeting activated host T, NK cells	Only target activated lymphocyte, spare resting cells	Potential resistant	(90, 91)
Immune synapse disruption	Dissemble of immune synapse	New layer of stealth	None reported	(93, 95)

HLA, human leukocyte antigen; ICI, immune checkpoint inhibitor; ADR, alloimmune Defense Receptor.

#### 3.2.1 Global disruption of HLA molecules: B2M KO, TAPi

HLA class I contains the polymorphic HLA-A, HLA-B, and HLA-C surface proteins. These molecules are heterodimers that consist of two polypeptide chains: the polymorphic HLA-encoded alpha chain and B2-microglobulin (B2M). HLA class I molecules are expressed in all nucleated cells and are the anchors to present intracellular peptides to CD8+ T cells (20). Direct targeting of the specific alpha chain HLA-A evades the immune response (67). The knockout of B2M to achieve reduced allorecognition by CD8+ T cells is by far the most common strategy in allogeneic cell transplantation and was first described in the stem cells (68–71), a finding which has been shown in iPSC derived cells (72, 73) In T cell therapies, HLA class I disruption is often combined with disruption of the TRAC locus to prevent surface expression of the T cell receptor (TCR) and preclude GvHD (74, 75).

Reducing HLA class I expression (via B2M KO) is not sufficient for full stealth, as HLA class I molecules have an important function as inhibitory ligands for NK cells. HLA-C and certain HLA-A and HLA-B alleles are ligands for Killer-cell immunoglobulin-like receptors (KIRs). HLA class I interactions with KIRs and NKG2A/CD94 play a major role in self-tolerance of NK cells, such that when these interactions are lost, the balance between activating and inhibitory signals on NK cells is shifted towards activation, resulting in a “missing-self” lysis of target cells (76). Expression of synthetic HLA-E–or HLA-G- β2m fusion proteins on target cells suppresses activation of NK cells that express the inhibitory receptor heterodimer NKG2A–CD94 (77, 78). An alternative approach to avoid CD8+ T cell HvG while minimizing the induction of NK “missing-self” is to specifically delete the HLA-A, HLA-B, and HLA-C genes while leaving B2M and HLA-E intact to engage NKG2A on patient NK cells (72).

HLA class II is frequently knocked out on the surface of adoptive cells in order to elude recipient CD4+ T cells. Major or small mismatches with HLA class II during an allogeneic encounter activate CD4+ T cells, which then promote allo-reactive CD8+ T cells and cause direct cytotoxicity from CD4+ T cells. A master regulator of MHC-II (major histocompatibility complex II) expression, the transcription factor class II major histocompatibility complex transactivator (CIITA) decreases surface expression of HLA class II when CIITA is knocked down (79). CIITA-KO hypoimmunogenic iPSC lines have been produced by a number of research groups employing CRISPR technology, either alone or in conjunction with B2M-KO (72, 73, 80, 81). A cell therapy lacking both HLA class I and II (B2M KO plus CIITA KO) will be required to prevent host CD8+ and CD4+ T cell responses and avoid any potential donor HLA antibodies the patient may produce or have already developed.

Another strategy for avoiding immune recognition is based on decreasing expression of MHC molecules and the antigen processing and presentation machinery (APM), including latent membrane protein (LMP) 2 and LMP7, transporter associated with antigen processing (TAP) protein. This is a mechanism by which malignancies are known to escape immune recognition (82, 83). These molecules mediate and regulate efficient antigen processing and presentation; subsequent T-cell responses have been abolished by shRNA-mediated TAP1 knockdown in combination with CIITA depletion in engineered adoptive cells (84). In addition, genetic disruption of TAPBP1 significantly reduced immunological rejection in mice (85).

#### 3.2.2 Harnessing immune checkpoints: CD47, PD-L1, CTLA4

Another strategy to prevent HvG is to overexpress CD47 (80). CD47 is a transmembrane protein with a well-described role as a “don’t eat me” signal due to its binding to signal regulatory protein a (SIRPa) on myeloid cells (86) and high CD47 expression on tumor cells is thought to protect tumor cells from immune responses (87). Recently, it was found that IL-2 stimulated NK cells upregulate SIRPa and can be inhibited through high levels of CD47 expression on B2M-KO target cells (81). PD-L1 overexpression alone or in combination with CTLA-4 was shown to improve iPSC-derived islet function and persistence of in a humanized mouse (88, 89). Altogether, the concept to express ligands for inhibitory receptors is a promising strategy to evade patient NK cells for T cell-based therapies, however for NK cell therapies, a careful evaluation is necessary to ensure that trans inhibition does not limit their function.

#### 3.2.3 Targeted killing of alloreactive cells: alloimmune defense receptor

Following initial stimulation, T cells and NK cells upregulate surface expression of several costimulatory molecules of the tumor necrosis factor receptor (TNFR) family, including CD27, 4-1BB, OX40 and CD30. These TNF family receptors are markers for activated lymphocyte populations, distinguishing them from naive populations (90). A new synthetic receptor called alloimmune defense receptor (ADR) selectively recognizes 4-1BB. The ADR-expressing T cells resist cellular rejection by targeting alloreactive lymphocytes *in vitro* and *in vivo*, while sparing resting lymphocytes. Cells co-expressing CAR and ADR persisted in mice and produced sustained tumor eradication in two mouse models of allogeneic T-cell therapy of hematopoietic malignancies and solid tumors. This approach enables generation of rejection-resistant “off-the-shelf” allogeneic T-cell products to produce long-term therapeutic benefit in immunocompetent recipients (91, 92).

#### 3.2.4 Disruption of immune synapse

Immune synapse formation is required for NK cell and T cell cytolytic function and enables the precise delivery of lytic-granule contents onto a susceptible target cell. Immune synapse formation is mediated by binding of the adhesion molecules CD54 (also known as ICAM-1) and CD58 (also known as LFA-3) on target cells to their cognate receptors, the integrin CD11a/CD18 (also known as LFA-1 or ITGAL) and adhesion and costimulatory receptor CD2 on immune cells (93, 94). Recent combined genetic deletion of CD54 and CD58 had shown significantly better *in vivo* persistence compared to both B2M -/- CAR T cells and HLA-E +B2M -/- CAR T cells in the presence of PBMC from healthy donors (95).

Overall, there are many proposed strategies to avoid the HvG response, and their efficacy will need to be determined in clinical trials. Importantly, better characterization of patient immune responses against administered allogeneic NK or T cell therapies will facilitate improved stealth approaches in the future.

### 3.3 Avoid GvHD: TRAC KO or choose an antigen specific TCR

Following allogeneic T cell transplantation, GvHD still presents a challenge for successful treatment. When immunocompetent donor T cells identify the recipient host as alien, they initiate an immune response against allogeneic antigen-bearing cells, which results in the death of host tissues and the development of GvHD. Even with current preventive measures, GvHD patients still have high rates of morbidity and mortality, with only about 40% of patients having a durable response to corticosteroid therapy (96, 97). Knocking out the TCR alpha chain is a strategy that has been explored to prevent GvHD. This approach involves genetically modifying donor T cells so they lack the alpha chain of the TCR, which is necessary for the recognition of host antigens and the subsequent immune response that leads to GvHD. Briefly, T cell activation is dependent on antigen recognition by the TCR. The TCR is composed of multiple subunits, including the alpha and beta chains. By knocking out the alpha chain, the TCR is rendered non-functional, which means the T cells cannot effectively recognize and attack the host tissues. This could potentially reduce the incidence and severity of GvHD after allogeneic cell transplantation. Multiple studies have generated CAR T cells with disrupted endogenous TCR to avoid the risk of GvHD inherent in allogeneic T cell therapy (98–100).

### 3.4 CAR insertion for specific targeting and hnCD16

CARs are synthetic receptors that redirect lymphocytes to recognize and kill cells expressing the target ligand. CARs have a modular design with four major components: an extracellular antigen-binding domain, a hinge, a transmembrane domain and an intracellular signaling domain (101). Variation of each of these component parts of CAR constructs enables fine tuning of the functionality and anti-tumor activity of the resultant CAR T cell product and has the potential to improve the safety and efficacy of CAR-T-cell therapy (102). For this purpose, several CAR generations have been generated, and the fifth generation is currently being tested in clinical trials (103). Recently, CAR gene constructs have been modified to express an ‘armoring’ protein, such as IL-12 or IL-15, to enhance T or NK cell function (101).

CAR-engineered T cell therapeutics were the first to emerge, demonstrating impressive clinical results, resulting in FDA approvals for hematological malignancies (1). CARs conventionally designed for T cells with CD3 zeta and T cell co-stimulatory signals have also been used for generation of CAR NK cells, and studies have demonstrated that these cells can effectively and specifically target tumors, while maintaining a desirable safety (11). Despite intensive research efforts to define optimal CAR design for both T and NK cells, a universally improved CAR structure has not yet been identified. As of now, each CAR construct needs empirical testing for evaluation, and several studies indicate that small modifications can have major consequences on the therapeutic outcome.

NK cells express the activating immunoglobulin gamma Fc receptor CD16a, which identifies the Fc portion of IgG antibodies attached to target cells. Patients treated with the high affinity CD16 variant (F158V) and monoclonal antibody have demonstrated enhanced antitumor responses (104). A second Fc receptor, CD64 binds to IgG with 30-fold greater affinity than CD16. iNKs expressing the fusion receptor of CD64 extracellular binding region and CD16a transmembrane, intracellular domain killed cancer cells effectively when combined with anti-HER2 trastuzumab or anti-EGFR1 cetuximab antibody. The higher affinity of CD64 allowed for monoclonal antibodies to be pre-adsorbed to the NK cells expressing the recombinant CD64 and improved tumor targeting without additional antibody (105).

### 3.5 lymphodepletion conditioning: CD52 KO for CD52 Ab

Lymphodepletion is a necessary process for patients undergoing hematopoietic stem cell transplants in order to make space for the transplanted cells. They currently need intense, non-specific lymphodepletion either using ionizing radiation or cytotoxic agents such as fludarabine and cyclophosphamide (flu/cy). Replacing non-specific lymphodepletion with targeted antibody-based conditioning could avoid harming mature hematopoietic cells and result in significantly less inflammation and unintended collateral organ damage than existing conditioning regimens. One way to improve therapeutic efficacy is through recurrent antibody-based infusion and conditioning after transplanted cells’ cell surface antigens have been selectively eliminated.

CD52 is highly expressed on mature T and B lymphocytes, with lower expression on other blood cells, such on monocytes, macrophages, and natural killer cells. Importantly, CD52 is not expressed on CD34+ HSCs. Therefore, the use of a depleting anti-CD52 antibody would specifically target lymphocytes of interest (B and T cells), while sparing the critical HSCs, required to repopulate the blood compartment. In support of this, a homozygous CD52 knockout mouse revealed no significant difference on lymphocyte populations, including resting T- and B-cell numbers (106). Thus, CD52 is an ideal target to knockout in donor cells in combination with antibody conditioning. Alemtuzumab is an FDA-approved, humanized mAb against CD52, which can effectively deplete T- and B-cell lymphocytes (107). The knockout of non-essential gene CD52 in combination with an FDA approved antibody is expected to be an effective combination to avoid current non-specific lymphodepletion conditioning regimens.

### 3.6 Novel strategies for efficient iT, iNK differentiation: inducible NICD, EZH1 KD, Flt3L and BMP4 KI

Current manufacturing of autologous CAR-T cell therapies transforms the patients’ own T cells, and this personalized manufacturing process adds significant cost, time and variability in producing the final T cell product. Building more robust, scalable, and reproducible manufacturing workflows for T cell therapies will help improve product safety and efficacy and will expand access to these life changing therapies. A major bottleneck on the path to T cell production is to efficiently differentiate hPSCs into definitive hematopoietic progenitors with T cell potential. Definitive hematopoietic stem cells (HSCs) arise from a cell type known as HE in a process called the endothelial-to-hematopoietic transition (EHT).

Prior approaches to generate T lineage-competent HPCs from hPSCs include the use of DL4-expressing immortalized stromal cell lines, or more recently developed cultures were coated with DL4, VCAM1, or DL4 coated microbeads (44, 45, 50). Notch signaling plays a pivotal role in the differentiation and development of lymphocytes, which are critical components of the immune system. There are four Notch receptors (Notch1–4) that interact with various ligands like Delta-like and Jagged proteins. These interactions initiate the Notch signaling cascade. Notch signaling is essential for T cell development in the thymus. It influences the fate of thymocytes, guiding them to differentiate into various T cell subsets. Dependent on Notch signaling, HPC differentiate into CD5CD7 double positive T cell progenitor cells. Under reduced Notch signaling and increased TCR signaling, CD4CD8 double positive cells are produced and mature to single positive T cells. To differentiate iT cells without notch ligand expressing feeder or DL4-VCAM1 coating and simplify the manufacturing process, an inducible expression of notch intracellular domain and VCAM1-VLA4 fusion can be knocked into the iPSC genome (**Figure 2**), which is turned on during the transition of CD34+CD45+ HE to CD5+CD7+ T cell progenitor cells and turned off after for T cell maturation.

Recent studies have revealed key roles for epigenetic regulators during definitive hematopoiesis and lymphoid development. The Daley group discovered that during embryonic hematopoietic development, EZH1, a part of polycomb repressive complex 2 (PRC2), is a crucial negative regulator of definitive lymphoid commitment (108). The production of CD3+ T cells was significantly increased in a subsequent study that used shRNA-mediated EZH1 knockdown or a doxycycline-inducible CRISPR interference (CRISPRi) construct into iPSC-derived CD34+ HE cells to transcriptionally repress EZH1 expression. This was due to the depletion of EZH1 during T cell specification (week 0-2) (45). EZH1 knockdown T cells displayed a significant increase in CD3+TCRαβ+ and decrease in CD3+TCRγδ+ T cells, indicating that EZH1 knockdown promotes differentiation towards αβ T cell fate rather than γδ T cells and exhibits a more mature T cell phenotype, highly diverse T-cell receptor (TCR) repertoire and enhanced antitumor activities. EZH1 is a positive regulator of Notch signaling, and this study further emphasizes the importance of timing and strength of notch signaling during T cell development (45). Knockin of the tunable EZH1 expression elements in iPSC could further improve the potency and yield of iT.

Further an elegant study from Zandsra group demonstrated the engineering of iPSCs with tunable cytokine signaling (109). The result was the precise control of the differentiation outcome and complete elimination of the need for exogenous BMP4 by engineering stem cells to express and secrete BMP4, a factor that is typically added exogenously to promote germ-layer differentiation or by using synthetic microRNA to fine-tune BMP4 expression level (110, 111). FLT3L is also essential for T cell development and commonly supplemented in the differentiation process. Integration of genetic codes to control BMP4 and FLT3L expression will likely be the subject of future engineering.

### 3.7 Increase iT stemness: IL-7RF, FOXO KI

The effectiveness of T cell therapy is limited by the fast exhaustion and death of T cells. Therefore, one strategy to improve the efficacy of T cell therapies is to increase the cells’ “stemness,” or their capacity to self-renew and differentiate into distinct kinds of T cells. T cell stemness is heavily dependent on the interaction between IL-7 and its receptor IL-7R. In fact, according to analyses using the PanCancer TCGA and iATLAS datasets, RNAseq of bulk tumors and scRNAseq of TILs, gene expression of IL-7R pathway components on tumor bulk is strongly connected with improved overall survival (OS) or progression-free survival (PFS) across various malignancies (112). In addition, expression of IL-7R and/or the IL-7R pathway is significantly greater in immune checkpoint inhibitors (ICI) responders versus non-responders in melanoma, NSCLC, ovarian, TNBC, HNSCC, and/or kidney malignancies (112). Genes associated with stemness are upregulated in TILs overexpressing IL7R, while genes associated with exhaustion are downregulated. Furthermore, IL-7R high TILs express a significantly higher quantity of BCL2, an anti-apoptotic molecule, and are less apoptotic.

Another major limitation of CAR T cell therapies is the poor persistence of these cells *in vivo*. The expression of memory-associated genes in CAR T cells is linked to their long-term persistence in patients and clinical efficacy. Recently, two separate research teams have found another way to rejuvenate these cells: make them more like stem cells. Taking into account that IL-15 improves CAR T cell persistence and metabolism, Chan et al. set out to discover important transcription factors that are elevated by this treatment. A Foxo1 gene signature was found to be significantly enriched when the epigenome and transcriptome of CAR T cells grown in the presence of IL-15 were analyzed (113). In CAR T cells generated from either healthy human donors or patients, overexpression of a constitutively active form of FOXO1 (FOXO1-ADA) promotes a stem-like phenotype, which corresponds with increased mitochondrial fitness, durability, and therapeutic efficacy *in vivo*. Doan et al. discovered that endogenous FOXO1 gene editing or pharmacological suppression reduced memory-associated gene expression, induced a state resembling tiredness, and decreased CAR T cell antitumor activity. In environments of prolonged stimulation, CAR T cells that overexpressed FOXO1 maintained their functionality, memory capacity, and metabolic fitness. They also demonstrated improved durability and tumor control *in vivo* (114). Thus, these findings provide an engineering strategy with translational potential to enhance the effectiveness of CAR T cells against solid tumors by genetically enforcing a favorable metabolic phenotype.

Numerous other strategies have been investigated to positively influence the development of CAR T cells. These include the overexpression of additional transcriptional regulators IRF4, c-Jun (115, 116), the application of homeostatic cytokines by tethered IL-15 (117), and epigenetic regulation with BET bromodomain inhibitors (118), which has emerged relatively recently.

### 3.8 Increase iNK *in vivo* persistence: IL-15RF KI, CISH KO

IL-15 plays a crucial role in the differentiation and survival of NK, it promotes the survival of these cells by maintaining the expression of anti-apoptotic factors like Bcl-2 (119). Furthermore, NK cells require IL-15 to mature in order to become responsive to this cytokine and reach functional maturity (120). Greater *in vivo* expansion and longer-term persistence are induced in CB-NK cells transduced with a fourth-generation vector encoding anti-CD19 CAR and IL-15 compared to nontransduced (NT) NK cells (121).

A key negative regulator of IL-15 signaling belongs to the suppressor-of-cytokine signaling (SOCS) family of proteins, known to play a significant role in NK cell biology (122, 123). One of its components, the CISH gene, is a key negative regulator of IL-15 signaling; it encodes the cytokine-inducible Src homology 2-containing protein (CIS) (124, 125). CISH knockout iPSC-NK cells have improved expansion, enhanced anti-tumor activity, and persistence *in vitro* and *in vivo*, and have exhibited improved metabolic fitness, which is mediated by the mTOR signaling pathway (126). CISH gene knockout in IL-15–secreting CAR-NK cells could improve their metabolic fitness, permitting greater *in vivo* persistence and cytotoxic function (127). When IL-15 expression is coupled with the disruption of the cytokine checkpoint gene CISH, CAR-NK cells’ therapeutic potential may be significantly amplified in the clinic.

### 3.9 Increase metabolic fitness: CD38, mbIL21 KI

CD38 is an enzyme with NAD+ glycohydrolase and its expression can lead to NAD+ depletion and immune cell exhaustion. CD38 is a multifunctional ecto-enzyme that plays a role in metabolism by metabolizing NAD+ and mediating nicotinamide dinucleotide (NAD+) and extracellular nucleotide homeostasis, as well as intracellular calcium signaling (128). CD38 knockout has been studied for its potential to increase metabolic fitness. Knocking out CD38 in mice can lead to increased longevity and protection against the development of cancers, especially under high metabolic pressure, such as high-fat diets (129). Additionally, CD38 knockout NK cells have shown increased resistance to oxidative stress-induced death and enhanced metabolic fitness due to a reduction in reactive oxygen species (23, 130). This suggests that CD38 knockout can be beneficial in improving metabolic fitness and may have therapeutic potential in conditions associated with metabolic dysfunction of NK or T cell therapy (131).

Members of the common gamma-chain receptor family, IL-15 and IL-21, have all been shown to have well-documented effects on NK cells; IL-15 is essential for NK cell development but IL-21 can improve NK cell viability and functions with increased metabolic fitness (33, 132). While IL-15 primarily signals through STAT5, IL-21 is known to largely signal through the STAT3 component of the JAK/STAT pathway with minimal participation from STAT5. Human telomerase reverse transcriptase (hTERT) is known to be activated by STAT3 (133). NK-cell senescence from mbIL15-mediated expansion can be reverted through hTERT gene editing (134). NK cells expanded with membrane bound (mbIL-21) have longer telomeres and less senescence than those expanded with mbIL-15, mbIL-21 promotes improved proliferation of human NK cells (33). IL-21-expanded TILs exhibited a ‘young’ phenotype with longer telomeres and higher expression of CD27 and CD28, and this phenotype was linked to stem-cell-like improved lifespan (132, 135). mbIL-21 increased NK cell metabolism with a shift towards aerobic glycolysis, induced robust and sustained proliferation of highly cytotoxic NK cells which exhibit increased cytotoxic function against various cancer cells (34). Inducible expression of mbIL-21 at expansion stage of iNK production may provide large quantities and mature, potent cell products for clinical applications.

### 3.10 Increase homing/infiltration: chemokine receptor KI

One limitation on the efficacy of CAR T or NK cell therapy is insufficient homing to or infiltration of the relevant sites (e.g. tumor, BM, secondary lymphoid tissues). NK cells develop mainly in the BM and egress into the blood circulation when they mature. They then migrate to peripheral tissues, though some special subsets home back into the BM or secondary lymphoid organs (136–138). NK cell lineage comprises of remarkably diverse population, two major PB NK cell subsets are CD56^bright^ and CD56^dim^ (139, 140). Normally, BM, lung, spleen, subcutaneous adipose tissue, and breast tissue are dominated by CD56^dim^ NK cells. In contrast, the proportion of total NK lineage cells in the stomach and intestinal mucosa, liver, uterus, visceral adipose tissue, adrenal gland, and kidney is significantly enriched in CD56^bright^ NK cells (141, 142). Accordingly, these distinct patterns of tissue localization correspond with markedly different patterns of chemokine receptor expression. While both subsets of NK cells express CXCR4, PB CD56^bright^ NK cells express CCR7, CXCR3, high levels of L-selectin (CD62L), for homing and/or entry into tissues that are secondary lymphoid or expresses the reciprocal ligands. Conversely, PB CD56^dim^ NK cells express sphingosine-1-phosphate receptor (S1PR5), and CXCR1, CXCR2, and CX3CR1 (141, 143, 144).

CAR T or NK cell trafficking and retention within tumor sites is essential for optimal anti-tumor efficacy. NK cells’ migration and homing to the lymph node-associated chemokine CCL19 against hematological malignancies was improved by NK cells’ induction of CCR7 expression (145, 146). Enhancing CAR-NK cells targeting the glioma antigen epidermal growth factor variant III (EGFRvIII) with CXCR4 expression led to better chemotaxis for U87-MG glioblastoma cells. These cells release CXCL12/SDF-1α, a CXC chemokine that interacts with receptors CXCR4 and CXCR7 (147). A novel strategy to improve homing and target NK cell-based immunotherapies to the BM (critical for ablation of auto-reactive plasma cells or hematological malignancies) is the ectopic expression of CXCR4 gain of function mutant R334X on expanded NK cells, which led to significantly greater BM homing after adoptive transfer into NSG mice compared to non-transfected NK cell controls. Additionally, BM migration of CXCR4 knockin iNK cells targeting various antigens results in superior tumor cell killing in the marrow in aggressive disseminated heme xenograft models (148, 149). Furthermore, inducing expression of CXCR1 in NK cells with a NKG2D CAR were shown to significantly increase anti-tumor responses in subcutaneous and intraperitoneal xenograft models along with an intravenous injection model against established peritoneal ovarian cancer xenografts (150).

### 3.11 Overcome suppressive TME: decoy receptors KI, A2AR KO

The TME is made up of a harsh metabolic environment that is characterized by a variety of immunosuppressive metabolites, hypoxia, acidity, upregulation of transforming growth factor beta (TGFβ), and glucose and amino acid deprivation (151). These factors work together to impede efficient antitumor immunity and are likely responsible for the challenges in treating solid tumors with cell therapies.

Because of its suppressive role in the TME, TGFβ has been targeted to boost cell therapy anti-tumor response. TGFβ mediates downregulation of NKG2D, NKp30, TRAIL, and DNAM1 receptors on activated NK cells (152, 153). To shield adoptive NK cell therapies from the suppressive effects of TGFβ, introduction of a dominant negative form of TGFβ type II receptor (TGFβRII) efficiently blocked TGFβ signaling and maintained cell surface expression of receptors and cytotoxicity in NK and T cells (154–156). Elegant strategies embracing the inhibitory cytokine and converting it into a potent stimulatory signaling have been created by rewiring the recognition domain into a second-generation CAR-T cell to orchestrate upregulation of cytokine production (157). Similarly, expressing a CAR with a TGFβRII extracellular and transmembrane domains combined with the intracellular domain of NKG2D on NK-92 cells converted the immunosuppressive signal into increased cytotoxicity while preventing downregulation of NKG2D surface expression (158). This strategy has also been applied to other inhibitory receptors such as PD-1, generating a PD-1 CAR with NK tailored endodomains such as NKG2D or DAP10/NKG2D to mediate cytotoxicity by NK cells against solid malignancies in the TME (159, 160).

The A2AR (adenosine A2A receptor) knockout and its role in the suppressive TME is a significant area of research in cancer immunotherapy. CD39 and CD73, which are highly expressed in a variety of TME cell types, regulate the extracellular environment’s metabolism of ATP and adenosine (161). Tumor cell immune evasion and inhibition of antitumor immune responses are facilitated by the CD39/CD73/A2AR pathway, which plays a critical role in the formation of an immunosuppressive TME (162). Targeting the A2AR, either through genetic knockout or pharmacological inhibition, can improve the function of antitumor immune cells (163, 164).

### 3.12 Overcome the physical barrier of stromal cells

The extracellular matrix (ECM) of tumor stroma creates a physical barrier to cancer therapies by preventing infiltration of therapeutic agents into tumors. ECM is made up of various structural molecules such as fibrous proteins, glycosaminoglycans, and proteoglycans. These are produced by tumors and cancer-associated fibroblasts (CAFs) that contribute to tumorigenesis (144). Normal tissue has thin fibroblasts in elongated spindle shape. Fibroblasts are thought to be in a resting state most of the time, but they can become activated in response to stimuli including stress, hypoxia, and cytokines as well as tissue injury (165). Following the wound healing, the quantity of activated fibroblasts decreases, and they most likely return to their resting state (166). However, in tumors, fibroblasts are often hyper-activated through mediating factors such as TGF-β, platelet-derived growth factor (PDGF) and fibroblast growth factor 2 (FGF2). CAFs are therefore considered as an irreversibly activated heterogeneous population of fibroblasts with distinct functions (165). They greatly contribute to the TME’s immunosuppression by secreting various chemokines and cytokines, including TGF-β, IL-6, IL-8, IL-13, CXCL12, and VEGF (167).

Fibroblast activation protein (FAP) is a membrane protease that is highly expressed CAFs. By altering the ECM, FAP can modify the TME, and its overexpression on cancer reduces the effectiveness of CAR-T cell therapy in solid tumors and is linked to a poor prognosis in a number of malignancies (168). One tactic being investigated to overcome barrier stroma cells is targeting FAP using CAR-T cells. FAP-targeting CAR-T cells have been engineered to target CAFs in various solid cancers, such as mesothelioma, lung and pancreatic cancers (169). A number of studies have shown anti-tumor activity in preclinical models (170, 171). Anti-FAP CAR-T cell treatments have been tested in clinical settings. The effectiveness of CAR T cell treatment may be increased by targeting FAP in addition to cancer antigen targets.

## 4 New gene editing technologies make engineering of complex cell therapies possible

In the past decade CRISPR–Cas9-based technologies have revolutionized basic and applied research in biology. However current gene integration approaches require DNA double-strand breaks and rely on repair pathways such as homology directed repair (HDR) that are inactive in terminally differentiated non-dividing cells. Programmable and multiplexed genome integration of multi-kilobase DNA cargo is still challenging. Together with the new families of editing enzymes, which include transposases, integrases, recombinases and single-stranded DNA-annealing proteins, new CRISPR/Cas-based long sequence integration technologies have been developed. This discipline is rapidly evolving, and it has the potential to spark a new wave of ground-breaking biomedical applications. These new technologies are critical to the advancement of cellular therapies given the multitude of edits mentioned above that are needed to ensure safe and effective treatments.

Traditional CRISPR/Cas9 systems exploit one of three types of DNA repair mechanisms: HDR, nonhomologous end joining (NHEJ), and microhomology-mediated end joining (MMEJ). These strategies may result in imprecise insertions or deletions with substantial indel errors, and the efficiencies vary greatly depending on cell type. Recent new CRISPR techniques for multi-kilobase DNA cargo insertion have been developed to help overcome some of the limitations of previous editing technologies, including: (1) the transposon-encoded CRISPR/Cas system;(2) recombinase/integrase with CRISPR/nCas9; (3) single-stranded DNA-annealing protein (SSAP) editor coupled with CRISPR/dCas9 (**Table 5**).

**Table 5:** Summary of new gene editing technologies for precise large DNA insertion in human genome.

Enzymes	Technologies	Insert size	Efficiency	References
Transposases	INTEGRATE	>10kb	1% (HEK293T)	(173)
	HELIX	>10kb	0.04%(HEK293T)	(175)
SSAP	REDIT	>2 kb	5% (ESC)	(176)
	dCas9-SSAP	>2kb	4% (ESC)	(177)
Recombinase/Integrase	eePASSIGE (PASSIGE/TwinPE)	<6kb	<4% (iPSC)	(179)
	I-PGI (PASTE)	Up to 36 kb	50-60% (iPSC)	(178, 180)

shCAST, CRISPR-associated transposase from cyanobacteria *Scytonema hofmanni*; INTEGRATE, Insert Transposable Elements by Guide RNA-Assisted TargEting; HELIX, HE-assisted Large-sequence Integrating CAST-compleX; SSAP, single-stranded DNA-annealing proteins; REDIT, RecT Editor via Designer-Cas9-Initiated Targeting; TwinPE, twin prime editing; eePASSIGE, evolved and engineered prime-editing-assisted site-specific integrase gene editing; PASTE, programmable addition via site-specifc targeting elements; I-PGI, integrase-mediated programmable genomic integration.

Among the instruments discussed above, CRISPR-associated transposons have mostly been examined in a limited range of prokaryotes (172–174). Recombination independent, multi-kilobase DNA insertions at RNA-programmed genomic sites are made possible by CRISPR-associated transposases (CASTs). However, substantial off-target integration and a transposition mechanism that produces a mixture of acceptable simple cargo insertions and undesirable plasmid cointegrate products limit the usefulness of type V-K CASTs (172–174). Another programmable RNA-guided transposon system, called Insert Transposable Elements by Guide RNA-Assisted TargEting (INTEGRATE), is capable of transposing cargo genes up to 10 kb in length to the human genome, however it lacks control over the orientation of insertion (173). Recently, the 5′ nicking capability required for cargo excision on the DNA donor was restored by engineering a nicking homing endonuclease fusion to TnsB (dubbed HELIX). HELIX allows for cut-and-paste DNA insertion with high homogenous insertion product purity and significantly higher on-target specificity than canonical CASTs. However, HELIX has very low efficiencies (<0.1%) in mammalian cells and requires at least 4 protein co-factors, indicating that more work is needed to fully realize their potential in genome engineering applications (175). While HELIX has been examined in several mammalian cell types, there isn’t any assessment yet available in iPSC.

A new cleavage-free gene editor dCas9-SSAP, which utilizes microbial single-strand annealing proteins (SSAPs) with catalytically inactive dCas9, promotes the integration of long sequences in mammalian cells (176). With an efficiency of up to 20%, it works well for inserting kilobase-scale sequences into human cell lines in therapeutic places like AAVS1. When compared to previous editing techniques that rely on single-strand nicks or DNA double stranded breaks, dCas9–SSAP produces nearly zero off-target mistakes while facilitating homology-mediated transgene insertion via non-cutting Cas9s (176, 177).

Programmable Addition via Site Specific Targeting Elements (PASTE) is a novel method that combines the programmability of Cas9 nickase, the writing ability of reverse transcriptase, and the size-agnostic DNA integration capacity of large serine integrases (LSI) to enable the integration of large pieces of DNA into specific genomic locations (178). This method avoids the generation of DNA double stranded breaks, which allows for efficient multiplexing. A similar method has been developed using an engineered integrase (eePASSIGE) that has shown low efficiency integration in iPSCs (~4% using 5kb cargo) (179).

Recently, Tome has developed an improved version of the PASTE technology, Integrase-mediated Programmable Genomic Integration (I-PGI). I-PGI is capable of efficient gene insertion in iPSCs cells, achieving >50% integration using nanoplasmid cargos. Tome has demonstrated the ability to multiplex integration of up to 4 inserts, and have integrated very large cargo (>31 kb) using adenovirus templates. For one of its iPSC derived therapeutic programs, Tome has demonstrated to ability to generate 3 knock outs and insert 12 kb of code in a single process (180). This capability to create complex cell therapies with relative ease and high efficiency opens the door to the development of highly editing cell therapies, including multiple CARs, safety switches, stealth, and other edits to increase *in vivo* persistence, cell fitness, and antibody mediated lymphodepletion.

In conclusion, the development of sophisticated iPSC-derived cell therapies involving extensive engineering has been made possible by these novel long-sequence integration technologies.

## 5 Future directions and concluding remarks

The field of immunotherapy is on the verge of a revolution, thanks to the advent of iPSC-derived immune cells and genome editing technologies. These cells have the potential to become the cornerstone of treatment for myriad diseases, heralding a new era in cell therapies. However, before this potential can be fully realized, several significant challenges must be addressed.

Optimization of Differentiation Protocols. The journey of an iPSC to a mature and functional immune cell is complex and requires precise control over the differentiation process. Current protocols need refinement to ensure that the resulting cells are not only mature but also possess the functional capabilities necessary to combat diseases effectively. This optimization is crucial for the cells to perform their intended therapeutic roles once administered to patients.

Minimizing Immunogenicity and GvHD. One of the primary concerns with off-the-shelf cell products is their potential to elicit an immune response in the recipient, leading to rejection or other adverse effects. Researchers are working to minimize the immunogenicity of iPSC-derived cells to ensure they can be used widely and safely across different patient populations.

Scalable Manufacturing Processes. To bring iPSC-derived immune cell therapies to the masses, it is imperative to develop manufacturing processes that can produce these cells in large quantities without compromising quality. Scalability is key to making these therapies affordable and accessible to all who need them.

Overcoming *In Vivo* Hurdles. Once inside the body, iPSC-derived immune cells face numerous challenges, including a suppressive TME, insufficient trafficking and infiltration, and the need for enhanced persistence and fitness to maintain efficacy. Researchers are exploring various strategies discussed in this review to help these cells overcome these hurdles and perform optimally *in vivo*.

Mitigating Safety Concerns. The potential for iPSC-derived cells to cause tumors or other safety issues is a significant concern that must be addressed. Ensuring the safety of these therapies is paramount to their clinical success and widespread adoption.

Advancements in Genome Editing Technologies. The use of advanced editing technologies offers the ability to integrate large DNA sequences at specific genomic locations in a multiplex fashion. This precision engineering of iPSCs and their derivatives at unprecedented speed and efficiency opens up the possibility of creating advanced immune cells obviating the obstacles of current immunotherapies.

Conclusion. iPSC-derived iNK and iT cells stand at the forefront of a promising new avenue in immunotherapy. These cells offer hope for treatment that could be applied to a wide range of diseases, from cancer to autoimmune disorders. As research continues and technology advances, iPSC-based approaches are set to revolutionize oncology and regenerative medicine, offering the potential for improved patient outcomes and a new standard of care in the years ahead.
